# The belief that certain illnesses can only be treated by traditional healers: a qualitative study exploring the ‘traditional illness’ Xifula in Limpopo, South Africa

**DOI:** 10.1017/neu.2026.10083

**Published:** 2026-04-30

**Authors:** Michael Galvin, Lezanie Coetzee, Patricia Leshabana, Nthabiseng Masebe, Aneesa Moolla, Peter Rockers, Denise Evans

**Affiliations:** 1 Department of Behavioral Health Science and Practice, University of South Florida, USA; 2 University of the Witwatersrand Johannesburg, South Africa; 3 Boston University, USA

**Keywords:** South Africa, global health, rare illnesses, cultural concepts of distress, psychosocial illness

## Abstract

**Background:**

In Africa, bewitchment is described as a moral framework that helps individuals and societies make sense out of disease and misfortune. Numerous African belief systems attribute difficult-to-treat health problems to bewitchment, rather than a conventional medical diagnosis, especially if biomedical doctors are unable to resolve the condition. This study examines one such illness, known as *xifula*, in rural Limpopo Province, South Africa.

**Methods:**

Using convenience sampling, 95 participants (≥18 years old) were interviewed to gauge their knowledge of the condition known as *xifula.* Data was analysed using NVivo software.

**Findings:**

*Xifula* is a cultural concept of distress related to bewitchment. The most common symptom of *xifula* is swelling of the legs or hands, followed by chronic headaches. Participants noted that *xifula* can start as a minor ailment, but then grows into a larger problem. After a long period without healing, however, *xifula* can begin to represent a significant threat to the individual’s health. Nearly all participants noted that *xifula* cannot be treated by Western biomedical professionals and instead requires a traditional healer to treat the condition.

**Interpretation:**

This research highlights the importance of context-specific education about the diagnosis and treatment of common ailments, as beliefs about afflictions, their causes, and appropriate treatments suggest a need for tailored information. As biomedical and traditional healthcare currently exist as parallel, siloed structures of diagnosis and treatment in Africa, there should also be efforts to bridge the divide between the two.

Significant outcomes

- While many studies have described forms of ‘traditional illnesses’ related to bewitchment in sub-Saharan Africa, very few have elucidated the details of specific forms of bewitchment in particular locales and their impact on health-seeking behaviours today.
- Second, this study highlights the ways in which biomedical and traditional forms of healthcare currently co-exist in this context, albeit generally in parallel, siloed structures of diagnosis and treatment.
- Our findings suggest, when added to the previous literature, that *xifula* continues to represent a significant explanatory model of illness in present day Limpopo Province, South Africa. Although some studies have described the phenomenon of *xifula*, few have examined this ailment as in-depth. By understanding how different societies conceptualise illness, we can better develop effective and sustainable localised healthcare.

Limitations

- This research used snowball sampling and is thus not intended to be representative.
- Additionally, only women caretakers were recruited for this study. Thus, it is unclear if a study that included men would report the same findings.
- Lastly, there may be a social desirability bias, where participants say what they believe staff want to hear, in order not to risk further involvement in subsequent waves of data collection.

## Introduction

Previous research in Africa has established that health beliefs and help-seeking behaviours are strongly impacted by local religious and cultural formulations (Hammond-Tooke, 1981; Semenya *et al*., 2012; Jors *et al*., 2015; Cooper, 2016). In addition, medical theories have long contextualised illness within the realms of spiritual and social causation across sub-Saharan Africa (Hammond-Tooke, 1989; Gyasi *et al*., 2016; Opoku *et al*., 2018).

Studies of traditional beliefs in sub-Saharan Africa often identify illness aetiology based on spirit possession and bewitchment (Ngoma *et al*., 2003; Falen, 2018). More specifically, bewitchment – imprecations sent by others with the intention to cause harm – is viewed as a common explanatory model of illness as confirmed by many studies in sub-Saharan Africa (Crawford & Lipsedge, 2004; Edwards, 2011; Sodi *et al*., 2011).

Bewitchment in Africa has been described as a moral framework that helps individuals and societies make sense out of disease and misfortune (Hammond-Tooke, 1981). This religio-cultural phenomenon has significant consequences for understanding illness, care-seeking behaviours, as well as treatment and eventual recovery in this context (Hammond-Tooke, 1989).

Although Western biomedical explanations for illness have slowly been incorporated into African health systems in the last century, the belief in supernatural powers as explanations for illness and misfortune remains widespread (van der Zeijst *et al*., 2021). Therefore, illnesses are often characterised as ‘traditional’ or ‘Western’ depending on their origin and their ability to be treated either biomedically or by local spiritual healers (Haram, 1991; Steen & Mazonde, 1999; Kovačič, 2020; Pemunta & Tabenyang, 2020).

Many studies have examined how conditions such as non-communicable diseases have been commonly ascribed to bewitchment (Carrazana *et al*., 1999; Ngoma *et al*., 2003; Sorsdahl *et al*., 2009). In many traditional African belief systems, difficult-to-treat health problems are often attributed to bewitchment, as biomedical doctors are often unable to efficaciously resolve such ailments (Ngobe, 2015; Louw & Duvenhage, 2016). This study examines one such cultural concept of distress (CCD), known as *xifula*, in Limpopo province, South Africa.

## Methods

Study background, sampling, procedures, and measures were described in a previous publication (Galvin *et al*., 2024; Galvin *et al*., 2025). This was a cross-sectional, qualitative study nested within a larger study, the Developing Belief Network (DBN) Study, which is conducting research across 16 countries and 50 distinct cultural and religious settings (Weisman *et al*., 2024). The data collected for this sub-study were part of the second wave of data collection conducted in Greater Tzaneen Municipality, Limpopo Province, South Africa, in 2024. The primary objective of the DBN study is to document the cultural influence on children’s (a) acquisition and development of religious knowledge and behaviours and (b) transmission of this knowledge and these practices to other individuals. Other questions the DBN study sought to answer include (1) How does the acquisition of religious cognition and behaviour vary within and between populations? and (2) How do processes of social learning support the development of religious cognition and behaviour?

The DBN study employed a snowball sampling strategy, in which the Tzaneen-based study team collaborated with community health workers at six local primary healthcare facilities participating in the DBN study to identify eligible participants. Later, the study team asked the enrolled participants if they knew anyone else who might be interested in participating in the study. If so, the study team obtained contact details and contacted potential study participants to assess their eligibility and willingness to participate in the qualitative interviews. Those eligible and willing were invited to participate and attend a scheduled study visit at the study office in Tzaneen.

Participants were included in this study if they were: 1) caregivers of children between 5 and 11 years of age; 2) 18 years or older; and 3) fluent in Xitsonga and Sepedi (a dialect of Northern Sotho). It is important to note that these eligibility criteria applied to the larger DBN study, and caregivers were co-enrolled in both the main DBN study and this sub-study. The interviews for both were combined, and therefore, participants were interviewed on a single occasion, saving time and resources for participants and the study team. The sample for this analysis was therefore a convenience sample of participants enrolled in the main DBN study. As these participants were taken from the larger DBN study, and since women are generally the primary caregivers across cultures, all participants were women.

Study visits were conducted at a private study office in Tzaneen, and participants were provided transportation to and from their homes. On the day of their visit, participants who were invited to participate in the study spoke with a member of the study team who explained the study and obtained written informed consent. Participants were asked to sign a study consent form as well as an audio consent form so that the interview could be recorded and later transcribed and translated. All communication and study documents were conducted in local languages (English, Xitsonga, and Sepedi) by a local study team who were trained on all study procedures, including participant confidentiality and informed consent.

Interviews took roughly 45 minutes to complete. Participants received a small reimbursement of R150 (approximately $10 USD) to compensate them for their time lost and any out-of-pocket expenses they may have incurred. They were also provided with refreshments during the visit.

Participants were asked to provide information about their age, monthly household income, employment status, education level, ethnic group, religious affiliation, marital status, and the number of children in their household. Participants were also asked if they knew of an illness known as *xifula* (in Xitsonga language) or *sefolwana* (in Sepedi language). It is important to note that these are two of several words in both languages that refer to malicious supernatural forces (Fortrell *et al*., 2012). As *xifula* is more commonly cited in the scientific literature, authors will use this term in the paper. If participants had heard of this illness, they were then asked how the illness is acquired and transmitted, how it affects sufferers, and what symptoms it causes. They were then asked how this illness could be treated, and whether it should be treated by traditional healers, biomedical doctors, or both. Lastly, participants were asked if they knew anyone who had suffered from this ailment and, if so, what they knew about those experiences. Saturation was assessed by systematically analysing data during collection, tracking code frequency to identify when new codes stop appearing, and ensuring that no new conceptual categories are emerging.

The questionnaire was originally developed in English, and all translations into Sepedi and Xitsonga were culturally validated by team members prior to incorporation into the questionnaire. Wording was maintained consistently across participants, and assessors asked semi-structured follow-up questions as needed. All interviews were audio recorded and transcribed. Three study enumerators reviewed all transcripts and translated them into English. The analysis utilised a directed content analysis method that combined deductive and inductive aspects of code development (Hsieh & Shannon, 2005). Analysis was performed using the constant comparative method in which data were constantly being analysed while being collected to continuously compare new information to identify patterns and refine concepts (Silverman, 2005). This iterative process helped in creating theories that are grounded in the data itself, rather than starting with pre-existing frameworks. Themes were developed and refined by the three study enumerators and study PI (MG) and lead researcher (AM), resulting in a final set of codes. Two coders ultimately fully assessed each transcript in NVivo. Following coding, coders met to resolve discrepancies and finalise codes. Main thematic codebook elements included identification of main elements composing *xifula*, causes of *xifula*, symptoms of *xifula*, and treatments of *xifula*, with subcategories of codes under each theme. The codes are presented as follows in the Results section below. Data was analysed using NVivo software. The survey data were collected and stored in REDCap, an electronic data capture tool hosted by the University of the Witwatersrand, to maintain confidentiality (Harris *et al*., 2019).

The study was approved by the Human Ethics Research Committee (Medical) at the University of the Witwatersrand, Johannesburg (Clearance number: M210914). All participants provided written informed consent to participate in the study and to have their interview audio recorded.

## Results

This study examined a population of rural women living near the city of Tzaneen. Ninety-five individuals (*n* = 95) completed the study interview and were therefore included in this analysis (see Table 1). Participants ranged in age from 21 to 69 years. The population of this study was predominantly poor, with 81% of households having a monthly income of less than 5,000 ZAR (approximately $275). Only 31% of participants were employed, although many had husbands who were employed. More than half (57%) had completed high school or a secondary school education. The ratio of Tsonga to Pedi participants was 3:2.

**Table 1.** Sample characteristics (*n* = 95)

	*N* (%)	Mean (SD)
Age (min/max: 21–64 years)	–	34 (10.4)
Monthly income in ZAR		
Less than 2,000	20 (21)	–
2,000 to 5,000	57 (60)	–
5,000 or more	18 (19)	–
Employment status		
Employed	29 (31)	–
Unemployed	65 (68)	–
Retired	1 (1)	–
Education		
No formal education	3 (3)	–
Primary	34 (36)	–
Secondary	54 (57)	–
University	4 (4)	–
Ethnic group		
Tsonga	58 (61)	–
Pedi	35 (37)	–
Other	2 (2)	–
Number of children (min/max: 0–7)	–	3 (1.4)
Marital status		
Single	50 (53)	–
Married	35 (37)	–
Divorced	7 (7)	–
Widowed	3 (3)	–

## What is *xifula*?

According to participants, *xifula* is an illness that is related to ‘bewitchment’. As one respondent says, ‘*Xifula* comes from witches at night. It can affect anyone, it just depends on the person trying to bewitch you’ (Participant 34). Others agreed, saying ‘*Xifula* is done by people, it is witchcraft (*vuloyi*)’ (Participant 16). This reference to bewitchment as ‘things that happen at night’ was commonly referred to by participants in both Sepedi (*dilo tša bošego*) and Xitsonga (*swilo swa vusiku*) throughout the interviews for this study. In addition, participants also frequently referred to bewitchment as ‘done by people’, referencing the idea that others are sending illness or misfortune via supernatural means according to traditional African religious practices (Participant 42).

## What are the causes of *xifula*?

The vast majority of participants interviewed identified the causes of *xifula* as a form of bewitchment that impacts the limbs – specifically, the hands and arms, or the legs and feet. Therefore, one contracts *xifula* by ‘walking or stepping on something’ or ‘handling money or other objects’. As these objects were often said to be placed in the individual’s path or handed to them with the intention of bewitching them, ‘it [*xifula*] almost always affects the legs or hands’, one respondent says (Participant 44). ‘I grew up knowing that there is *xifula*, seeing people’s feet getting swollen. They say people send it onto you by putting it where they know you’ll pass by so you can step on it’, another remarked (Participant 25). Another described how ‘sometimes when walking around in your yard you might suddenly feel a piercing pain in your body and you may start shivering, indicating you’ve been impacted by *xifula*’ (Participant 84). Secondarily, a few participants report that you can get *xifula* by ‘eating something’ which has been bewitched, as well. This can result in chronic headaches as a symptom of *xifula*, as described in the second account in Table 2. One participant even described stepping on a frog in his path as seen in the fourth account in Table 2.

**Table 2.** Additional accounts of participants who experienced close encounters with *xifula*

“This [*xifula*] is the thing that killed my father… When my dad had it, I was convinced it was polio because when you have polio, you get swollen. At home, I am the only one who goes to church. The others believe in traditional healers. When they found out [it was *xifula* and needed to be treated by a traditional healer] it was already too late because they had already taken him to the hospital” (Participant 25)
“I once had it on my head. I had a very painful headache. I went to the clinic and it didn’t go away. I ended up going to the prophets and they told me I had *xifula*… I had abnormal headaches, and even if I drank pills [painkillers], it didn’t stop… It was unusual” (Participant 33)
“My brother had *xifula* on his foot two years ago. His toe was as black as this chair… he went to an apostolic church where [prophets] used teas and wool until he was healed” (Participant 46)
“I once had it [*xifula*]. I stepped on a frog at night. From there I started feeling pain and developed swollen legs” (Participant 57)
“I once had it [*xifula*] on my finger. I was preparing food for Christmas, and while taking out the maize meal from the bucket, I felt something biting my finger. When I checked, there was nothing there. That’s when I started feeling pain and my finger began to swell” (Participant 61)
“My brother had it [*xifula*]. We took him to the hospital and he was treated, but he died. He didn’t want to go to a traditional healer because he was a pastor and he believed he could be healed through prayer and fasting” (Participant 79)

Nevertheless, as previously described, *xifula* most commonly impacts the limbs. As one respondent says, ‘a person is hit on the hand or foot. They [witches] put something in your path. It touches or scratches you, and the area becomes wounded. Some people get it by stepping on something, others get it by receiving money. Like cash in their hand… But you won’t be affected unless it [the bewitchment] is directed at you’ (Participant 19). This description highlights the way in which individuals are perceived to be targets for bewitchment by either placing an object in your path that affects the feet and legs, or given money which impacts the hands and arms. ‘They give it to you through money so that you take it in your hand, or they put it in your path and make you walk on it and you end up falling down; then it [*xifula*] shows when you start limping’ (Participant 20).

It is also important to emphasise that *xifula* is a targeted attack where only the intended victim can be affected. As one respondent describes, ‘*Xifula* only affects whoever they [the witches] have put it for. If they put something in the path of the intended victim and they stepped on it, then the leg can get swollen and rot and have unusual wounds’ (Participant 39). In this sense, *Xifula* is not so much an illness that anyone can get by coming into contact with it, but rather the individual who contracts it is the victim of a targeted attack.

## What are the symptoms of *xifula*?

Most participants reported that the most common symptom of *xifula* is swelling of the legs or hands. The next most commonly reported symptom is a wound or rash on the legs or hands that won’t heal. ‘*Xifula* wounds do not heal, they always cause pain’, one respondent said (Participant 27). ‘You realise what it is when the wound doesn’t heal’, another remarked (Participant 48). This ability to identify *xifula* by a wound that will not heal is a key characteristic of bewitchment, according to many participants. ‘It is something that always causes pain on the body, it can be on the hand on the foot and it always causes pain that never goes away. Sometimes it can be a burning sensation and you have to put the foot or hand in cool water to calm down the pain. The main factor is that the pain never goes away’, according to one respondent (Participant 78).

There were a large variety of descriptions of symptoms of *xifula*, usually as a rash, a wound, a boil, or swelling of the limbs. As one respondent described, ‘if my leg is affected it may get swollen, change colours, and eventually become black’ (Participant 62). Participants noted how often *xifula* can start as a minor ailment, but then grows into a larger and larger problem. ‘It [*xifula*] starts as a small wound next to a nail on the thumb. You will experience a painful swelling in the thumb which does not heal’, one person said (Participant 40). After a long period without healing, however, *xifula* can begin to represent a significant threat to the individual’s health. ‘If it is on your hand it can destroy your hand’, another respondent remarked (Participant 38). One respondent noted how he once had *xifula* in his ‘private parts’, remarking ‘I couldn’t sit, it hurt a lot… for this reason a lot of people keep quiet about it’ (Participant 87). Several participants noted *xifula* can ultimately result in death if not treated in a timely and appropriate manner, as seen in the sixth account in Table 2.

## What is the treatment for *xifula*?

Nearly all participants noted that *xifula* cannot be treated at the hospital or by Western biomedical professionals. Rather, *xifula* requires a traditional healer to treat. While some respondents say that prophet healing or praying at church might help, the vast majority emphasised that African traditional healing is the primary means of curing *xifula*. As one said, ‘these are traditional supernatural things that they [witches] use to make people sick… the Western doctors won’t see anything when you are consulting with them’ (Participant 41). Another respondent noted how doctors can also misdiagnose *xifula*: ‘doctors might tell you that you have diabetes, whereas it is really *xifula*’ (Participant 35).

Some participants insisted that *xifula* ‘is not treatable at the clinic’ (Participant 35), or argued that ‘if you consult a doctor, it [*xifula*] won’t heal’ (Participant 47). Others claim that consulting a doctor may result in *xifula* that appears to improve, but the improvement will not last: ‘someone can go to the hospital now, then feel better at first, but then later get worse. The hospital doesn’t help’ (Participant 36). Yet, others argued that ‘going to the hospital makes it [*xifula*] worse,’ sometimes even resulting in the death of the patient (Participant 52).

However, if patients seek treatment from a traditional healer, the *xifula* can be cured. ‘If you go to the clinic, you will not improve, but if you go to the traditional healer, you become better’, claimed one (Participant 55). While some respondents claimed that prophets can also heal *xifula*, as seen in accounts 2 and 3 of Table 2, nearly all emphasised the importance of staying away from Western medicine. As one respondent stated, ‘prophets can use wool to tie around your hands and legs. They can also use their teas. But it cannot be healed the Western way’ (Participant 74).

## Discussion

There is a small but growing scientific literature examining the relationship between traditional African beliefs and illness in sub-Saharan Africa (Patel, 1995; Sylvia, 2000; Azevedo, 2017; Anizoba, 2021). The primary goal of these studies is to understand how ‘folk beliefs’ influence illness causation as well as decision-making processes for treatment (Golooba-Mutebi & Tollman, 2007a). As these concepts often do not feature in policy discussions, it is important to highlight their significant continued impact on pathways to healthcare in sub-Saharan Africa.

*Xifula*, as a particular diagnosis in the Xitsonga language, falls under a broader category of bewitchment, which has many translations in Xitsonga including ‘ku dlukula’, ‘xifulana’, ‘ku wutla rigadyi’, ‘khubalo’, ‘xidyiso’ or ‘xifula’ (Fottrell *et al*., 2012). In English, other translations might include words such as ‘witchcraft’, ‘evil spirit’, or ‘devil spirit.’ One study examining *xifula* in Limpopo province, South Africa found similar findings to this study (Stadler, 1996). Witches often ‘send’ misfortune by burying something under the entrance to someone’s house, which results in a boil and later a small sore appearing on the ankle or leg. Stadler describes how informants were sure they had been bewitched once the sore proved incurable by doctors at the clinic or hospital. Similar to this study’s findings as well, without appropriate treatment *xifula* could result in permanent disability or death.

Another more recent study in Limpopo examined patients suffering from leg paralysis and the perception of it being caused by *xifula* (Boene *et al*., 2020). In this study, participants described how *xifula* was often attributed to bewitchment caused by family members, such as in-laws. One woman with drop-foot described how her *xifula* was caused by her sisters-in-law and was currently being treated by traditional healers who rubbed herbal remedies on fresh razor blade cuts on her skin.

A third study examining *xifula* in Limpopo similarly recounts how patients contracted it by jumping over or stepping on ‘medicines laid along a path or at the entrance to a homestead by a witch’ (Golooba-Mutebi & Tollman, 2007a, p. 66). These medicines are referred to as *xidyiso,* which once ingested, turns into a live organism that eats the victim from inside the body. Authors found symptoms can include wounds and swelling of the limbs, in addition to severe head or stomach aches, and even stroke-like symptoms. In one case study, a young man repeatedly sought care from doctors without success. After visiting several traditional healers, he was ultimately cured by a local *inyanga* – or traditional herbalist – who was visiting his family at the time.

These studies therefore confirm many findings of this study, particularly that individuals with *xifula* are victims of bewitchment, which largely impacts the limbs via wounds, swelling, or paralysis. The participants in these studies also primarily sought care from traditional African healers. While these studies did not generally describe patients being successfully treated by prophets or other forms of Christian healing, future research could examine the relationship between traditional healers who use African versus Christian forms of healing for illnesses such as *xifula*.

While it is difficult to determine the precise extent to which *xifula* impacts modern-day populations in Limpopo province, folk beliefs concerning illness and its causation continue to significantly influence an individual’s decision about whether to select allopathic or traditional treatment. As symptoms of *xifula* often include various forms of bodily swelling and skin lesions – common symptoms of advanced stages of HIV/AIDS – some researchers argue that *xifula* might have seen a resurgence since the 1990s (Golooba-Mutebi & Tollman, 2007b). Others argue that growing populations and increased land conflicts may have resulted in increases of *xifula* historically (Gengenbach, 1998). Ultimately, however, anthropologists have found that traditional African explanatory models of illness, such as *xifula*, emphasise *why* something occurred rather than *how* – a perspective that Western medicine does not typically adopt – and may therefore remain relevant for this reason (Hammond-Tooke, 1981, 1989). It is important to note that this study does not adjudicate biomedical causation but rather documents beliefs and pathways to care.

*Xifula* could likely be characterised as a CCD as defined by the *Diagnostic and Statistical Manual* similar to others such as ‘Maladi Moun’ in Haiti which also refers to bewitchment, or illness sent by one person supernaturally with the intent to cause misfortune or harm (Galvin *et al*., 2023). Additionally, members of this study team have also conducted several studies regarding religio-cultural concepts of illness related to bewitchment in the South African context and may be relevant to readers of this study (Galvin *et al*., 2023a; Galvin *et al*., 2023b; Galvin *et al*., 2024a; Galvin *et al*., 2026)

Lastly, this study has several limitations. First, this research used snowball sampling and is thus not intended to be representative Additionally, only women caretakers were recruited for this study. Thus, it is unclear if a study that included men would report the same findings. Similarly, these findings from caregiver perspectives within the DBN recruitment rather than population beliefs. Lastly, there may be a social desirability bias, where participants say what they believe staff want to hear, in order not to risk further involvement in subsequent waves of data collection. However, it is not clear in which direction this would have impacted the data.

## Conclusion

This study examined the illness referred to as *xifula* among female caretakers in rural Limpopo province, South Africa. It found that *xifula* is an illness caused by bewitchment that primarily affects the limbs of the patient and is only treatable by traditional healing. This research, therefore, highlights the need for context-specific education about the diagnosis and treatment of common ailments, based on beliefs about afflictions, their causes, and appropriate treatments. As biomedical and traditional healthcare currently exist as parallel, siloed structures of diagnosis and treatment in Africa, there should also be efforts to bridge the divide between the two. Ultimately, understanding how societies interpret the essential factors that impact their health, as well as how health-seeking behaviours are influenced by local cultural notions and perceived aetiologies of illness, can help inform more effective, sustainable interventions and health promotion efforts.

## Data Availability

Deidentified data and other study information is available upon request from corresponding author with publication.
