# The belief that certain illnesses can only be treated by traditional healers: a qualitative study exploring the ‘traditional illness’ Xifula in Limpopo, South Africa—ERRATUM

**DOI:** 10.1017/neu.2026.10089

**Published:** 2026-07-17

**Authors:** Michael Galvin, Lezanie Coetzee, Patricia Leshabana, Nthabiseng Masebe, Aneesa Moolla, Peter Rockers, Denise Evans

**Affiliations:** 1 Department of Behavioral Health Science and Practice, University of South Florida, USA; 2 University of the Witwatersrand Johannesburg, South Africa; 3 Boston University, USA

The journal would like to apologise for the inclusion of errors as detailed below:

Page 1 Affiliation 1 ^1^Department of Behavioral Health Sciences, University of South Florida, USA should read ^1^Department of Behavioral Health Science and Practice, University of South Florida, USA.

The article has been corrected online.
